# 3D-Hydrogel Based Polymeric Nanoreactors for Silver Nano-Antimicrobial Composites Generation

**DOI:** 10.3390/nano7080209

**Published:** 2017-08-01

**Authors:** Albanelly Soto-Quintero, Ángel Romo-Uribe, Víctor H. Bermúdez-Morales, Isabel Quijada-Garrido, Nekane Guarrotxena

**Affiliations:** 1 Centro de Investigación en Ingeniería y Ciencias Aplicadas, Universidad Autónoma del Estado de Morelos, Cuernavaca 62209, Morelos, Mexico; soquia_17@hotmail.com; 2 Research & Development, Advanced Science & Technology Division, Johnson & Johnson Vision, Jacksonville, FL 32256, USA; aromouribe@gmail.com; 3 Centro de Investigación sobre Enfermedades Infecciosas, Instituto Nacional de Salud Pública, Dirección de Infecciones Crónicas y Cáncer, Avenida Universidad No. 655, Cerrada los Pinos y Caminera, Colonia Santa María Ahuacatitlán, Cuernavaca 62100, Morelos, Mexico; vbermudez@insp.mx; 4 Instituto de Ciencia y Tecnología de Polímeros, Consejo Superior de Investigaciones Científicas (ICTP-CSIC), c/ Juan de la Cierva, 3. E-28006 Madrid, Spain

**Keywords:** smart-hydrogel, polyurethane networks, thiol-acrylate networks, silver-hydrogel nanocomposites, Ag nanoparticles, antibacterial materials

## Abstract

This study underscores the development of Ag hydrogel nanocomposites, as smart substrates for antibacterial uses, via innovative in situ reactive and reduction pathways. To this end, two different synthetic strategies were used. Firstly thiol-acrylate (PSA) based hydrogels were attained via thiol-ene and radical polymerization of polyethylene glycol (PEG) and polycaprolactone (PCL). As a second approach, polyurethane (PU) based hydrogels were achieved by condensation polymerization from diisocyanates and PCL and PEG diols. In fact, these syntheses rendered active three-dimensional (3D) hydrogel matrices which were used as nanoreactors for in situ reduction of AgNO_3_ to silver nanoparticles. A redox chemistry of stannous catalyst in PU hydrogel yielded spherical AgNPs formation, even at 4 °C in the absence of external reductant; and an appropriate thiol-functionalized polymeric network promoted spherical AgNPs well dispersed through PSA hydrogel network, after heating up the swollen hydrogel at 103 °C in the presence of citrate-reductant. Optical and swelling behaviors of both series of hydrogel nanocomposites were investigated as key factors involved in their antimicrobial efficacy over time. Lastly, in vitro antibacterial activity of Ag loaded hydrogels exposed to *Pseudomona aeruginosa* and *Escherichia coli* strains indicated a noticeable sustained inhibitory effect, especially for Ag–PU hydrogel nanocomposites with bacterial inhibition growth capabilities up to 120 h cultivation.

## 1. Introduction

To date, many advanced infection treatments have been developed based on the discovery of new families of broad spectrum antibiotics. Nevertheless, the intense abuse and misuse of them makes unfeasible so far to ward off the increasing resistance of bacteria strains. Additionally, the declining interest in antibiotics research from the major pharmaceutical corporations [1] endangers the successful control of bacterial infections to address the challenge. Thus, efficient and innovative therapies against the emerging threat of resistant bacteria are highly desirable. In this respect, silver nanoparticles-based integrative solutions, due to their potential antimicrobial effects, could provide a compelling alternative [2,3,4,5]. Since ancient times, silver, in different forms, has been used as an antimicrobial agent against infections; thus, for more than 100 years, silver nanoparticles (AgNPs) have been used before recognizing their nanometric dimension [6,7]. More recently, the development of biomaterials based on polymeric hydrogels containing AgNPs is attracting significant interest. In particular, hydrophilic hydrogels are good matrices for the sustained dosage of silver ions (Ag^+^) also decreasing the toxicity of AgNPs [8,9].

Hydrogels are three-dimensional (3D) networks of hydrophilic polymers that swell in the presence of water; the versatility of these systems lies on the fact that by controlling chemical composition and crosslinking ratio, a wide range of materials can be modulated as function of the desired application [10,11]. Moreover, properties such as swelling degree and kinetics, stimuli responsiveness, or degradability can be tuned. In addition, their ability to absorb other substances makes them invaluable tools for the controlled dosage of drugs and other active substances [12]. Thus, nanohybrids generated by embedding AgNPs into hydrogels are of particular interest in medical treatments, for example preventing infection of topical wounds [8,13,14,15], because of their low toxicity, transparency to observe the wound, potential for extended release of drugs, and ability to keep the wound hydrated. Similarly, AgNP-hydrogel nanocomposites are being used as therapeutic contact lenses [16,17] for the treatment of fungal keratitis [16], as tissue engineering scaffolds [18] for bone replacement [19], and as colored cornea substitutes due to their anti-infective properties and potential for color modulation [20].

Several approaches for the synthesis of hydrogel nanocomposite materials with nanoparticles embedded in the hydrogel scaffold have been developed. Most of them involve a physical or chemical combination of independently generated NPs and hydrogels [18,21,22], or gel formation by mixing pre-synthesized NPs with polymers and gelator precursors [16,23,24]. Other strategies involved the in situ radical polymerization and reduction of AgNO_3_ during the hydrogel formation, mainly via the photochemical process [25,26]. Potential drawbacks of using these protocols are their synthetic complexity and the low control of the amount and distribution of particles formed within the 3D network.

As an alternative, in this study, we report a simple strategy to design two structurally diverse nanoparticle-hydrogel composites based on in situ reactive nanoparticle generation within a previously built hydrogels, by using two different reactive synthetic routes. So, while Ag-Thiol acrylate (Ag–PSA) nanocomposite generation involves a thiol-functionalized hydrogel matrix able to modulate the AgNP formation in the presence of a reducing agent; a new and improved protocol to synthesis nanosilver-loaded PU hydrogel (Ag–PU), with no presence of thiol functionalities neither external reducing agent, is performed. We tactically selected two series of hydrogels (thiol-acrylate (PSA) and polyurethane (PU)), as templates in the reactive synthetic approaches, and whose specific compositional structures satisfy the restrictive demand of biocompatibility and biodegradability; fundamental features for innovative biological applications [27]. In fact, both systems are mainly composed by poly(ethylene glycol) (PEG), a biocompatible hydrophilic polymer, and poly(ε-caprolactone) (PCL) which enables to modulate properties as viscoelasticity, hydrophobicity, shape memory effect, and degradability [28]. Furthermore, we took advantage of the synthetic protocols used in performing the 3D hydrogel networks to develop a novel and advantageous nanoparticle-formation procedure within the hydrogel. The yielded hydrogel matrices exhibit optical and swelling properties influenced by temperature and AgNP presence which last determine the Ag-nanocomposite efficiency on competitive and sustained antimicrobial properties. The effect of synthetic conditions and polymer composition in AgNPs uniformity and distribution together with the antimicrobial properties of the nanocomposites, on the basis of swelling-shrinking equilibrium and NP surface charge induced effect will be discussed in detail.

## 2. Materials and Methods

### 2.1. Materials

For thiol-acrylate (PSA) hydrogels preparation, poly(ethylene glycol) of average molecular weight (*M_n_*) of 3350 g·mol^−1^ (PEG3.3k), poly(ε-caprolactone) of *M_n_* 10,000 g·mol^−1^ (PCL10k), triethylamine (99%), acryloyl chloride (97%), pentaerythritol tetrakis(3-mercaptopropionate) (>95%), and 2,2-dimethoxy-2-phenylacetophenone (DMPA, 99%) were purchased from Sigma-Aldrich (St. Louis, MO, USA).

For polyurethane (PU) hydrogel synthesis, polycaprolactone diol (PCL_530_) *M_n_* (530 g·mol^−1^), poly(ethylene glycol) diol (PCL_600_) of *M_n_* (600 g·mol^−1^), and pentaerythritol ethoxylate crosslinker (PEG_300_) of *M_n_* (270 g·mol^−1^)—purchased from Sigma-Aldrich (St. Louis, MO, USA)—were vacuum dried at 80 °C for 24 hours before use. Stannous 2-ethylhexanoate (Aldrich, St. Louis, MO, USA, 92%) was used as received. Hexamethylene diisocyanate (HDI, Aldrich, St. Louis, MO, USA, 98%) was distilled before use, and 1, 2-dichloroethane (DCE, Aldrich, St. Louis, MO, USA, 99%) was dried by distillation from phosphorus pentoxide (Sigma-Aldrich, St. Louis, MO, USA, ≥98%).

Silver nitrate (AgNO_3_) and trisodium citrate dihydrate (NaC_6_H_5_O_7_·2H_2_O) were purchased from Sigma-Aldrich (St. Louis, MO, USA). The solvents chloroform (≥99.8%), ethanol (99.8%), *n*-hexane (≥99%), dichloromethane (DCM, ≥99%) from Sigma-Aldrich (St. Louis, MO, USA) and 2-propanone (Merck, Darmstadt, Germany, 99.8%) were used without previous purification. All aqueous solutions were prepared with ultrapure water purified with a Milli Q-POD water purification system (Millipore, Bedford, MA, USA).

### 2.2. Hydrogels Synthesis and Characterization

Both types of hydrogels used in this work were synthesized following existing protocols [29,30,31,32].

#### 2.2.1. Thiol-Acrylate Hydrogels (PSAs)

**Scheme 1:**
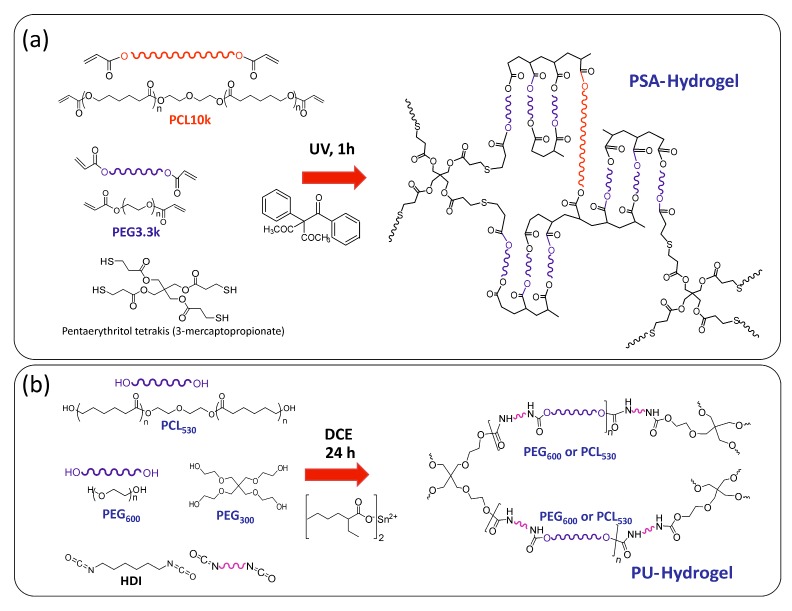
Schematic representation displaying the synthesis of thiol-acrylate (PSA) (**a**) and polyurethane (PU) (**b**) hydrogels, providing the polymeric chains throughout the hydrogel matrix for AgNP coordination and stabilization. The left-hand side of the scheme depicts more schematic versions of the polymeric chains, crosslinking agents, and catalysts used in the 3D-hydrogel matrix generation.

PSA molecular networks were synthesized by a two-step sequential procedure based on UV-initiated free radical polymerization of previously functionalized macromonomers (Scheme 1a). The macromonomer functionalization consisted of exchanging the end hydroxyl groups of PEG and PCL by alkene groups, as described by Kweon et al. [30] and Alvarado-Tenorio et al. [29]. Afterward, the chemically crosslinked PEG–PCL molecular networks were prepared via radical photopolymerization of thiols and acrylate alkene groups in the presence of the photo-initiator DMPA, under UV-irradiation at a wavelength of 365 nm and at room temperature for 1 h, using glass molds to obtain hydrogel films of 1 mm thickness.

As reported by Anseth and coworkers [32] these thiol-acrylate photopolymers polymerize using mixed-mode chain (acrylate homopolymerization) and step-growth (thiol-acrylate) competitive mechanisms, and their final network structures depend on the thiol/acrylate ratio. In the present work, all photopolymerizations were performed at a thiol/acrylate functional group ratio of 1/5 mol. Subsequently, a series of hydrogels were prepared by varying the concentration of PCL10k from 0 to 15%-mol. In addition, PEG3.3k and PCL10k homo-networks were also prepared using the respective acrylate macromonomers and tetrathiol, following the same procedure. The resulting gel sheets were removed from the glass plate, and 6 mm diameter uniform disks were punched out from each gel sheet using a stainless steel cork-borer. These hydrogel disks were immersed in DCM at room temperature for two days, in order to remove residual monomers. Finally, hydrogels were dried at room temperature until they achieved a constant weight.

#### 2.2.2. Polyurethane Based Hydrogels (PUs)

PU based hydrogels were synthesized by condensation polymerization between PEG and PCL diols, the diisocyanate HDI and PEG_300_ tetraol as crosslinker (Scheme 1b). All polymerizations were carried out using 5 g of macromonomers and 10%-mol of PEG_300_ crosslinker, the solvent DCE (40 wt %), the catalyst stannous 2-ethylhexanoate (0.3 wt %), and the diisocyanate HDI with a isocyanate/hydroxyl molar ratio of 1.05/1. The synthetic procedure was as follows: the oligomeric compounds were dried under vacuum at 80 °C for at least 6 h. Then, polyols were solved in DCE at room temperature and the diisocyanate and the catalyst were added under magnetic stirring. To obtain sheet shaped gels, the mixture solution was cast on a glass plate enclosed by a rubber framework-spacer with 1 mm thickness and sealed off with other glass plate in order to avoid air contact during the polymerization (24 h, 40 °C). Afterward, the gel sheet was removed from the glass plate and uniform disks 6 mm in diameter were punched out from the gel sheet using a cork-borer. In any case, disk-shaped hydrogels were immersed in ethanol for, at least, three days to remove the unreacted chemicals. During this time the solvent was replaced several times. After that, the hydrogels were dried at room temperature until constant weight was reached.

Both hydrogel types were characterized using a combination of attenuated total reflectance Fourier transform infrared spectroscopy (ATR-FTIR), thermogravimetric analysis (TGA), and differential scanning calorimetry (DSC). FTIR spectra were recorded on a Perkin Elmer Spectrum One spectrophotometer (Waltham, MA, USA) with the ATR technique (ATR-FTIR) and with a resolution of 4 cm^−1^. The thermal stability was determined from thermogravimetric analysis (TGA) with a TA Hi-Res TGA 2950 instrument (New Castle, DE, USA), at 10 °C·min^−1^, under 20 mL·min^−1^ of dry helium. The temperature at which the weight loss rate is maximum (*T*_max_) was determined from the peak maximum of the first derivative of the weight lost. The thermal transitions (glass transition (*T*_g_), endothermic crystallization (*T*_c_), and exothermic melting (*T*_m_) temperatures) were measured by means of differential scanning calorimetry (DSC) using a TA DSC Q100 apparatus (New Castle, DE, USA) connected to a cooling system to work at low temperatures. Samples were scanned at 20 °C·min^−1^ under 20 mL·min^−1^ of dry nitrogen from −75 to 150 °C. *T*_g_ values were determined in the second heating run cycle. Morphology of the lyophilized hydrogel samples was examined using a scanning electron microscope (SEM) Philips XL 30 (Eindhoven, The Netherlands) operated at 25 kV after coating the sample with Au-Pd (80/20) 10 nm film.

### 2.3. Ag-Hydrogel Nanocomposite Synthesis and Characterization

The loading of silver nanoparticles into the hydrogel networks was performed according to the following procedures.

#### 2.3.1. AgNPs within Thiol-Acrylate Hydrogels (Ag–PSA)

Dry pure hydrogel disks were swollen in an aqueous solution of AgNO_3_ (1 × 10^−3^ M) and sodium citrate (6.4 × 10^−4^ M) for 24 h at 4 °C. The aqueous solution containing silver salt loaded hydrogel disks was then placed into a silicon bath at 103 °C for 30 min to induce the reduction of the silver ions and the formation of AgNPs in the hydrogel structure. After 5 min, the colorless reaction mixture started turning yellowish, which further turned brown within 15 min, indicating the formation of AgNPs. The solution was then cooled down in a water bath.

#### 2.3.2. AgNPs within PU Hydrogels (Ag–PU)

AgNPs were incorporated into the structure of hydrogel after swelling pure hydrogel disks in an aqueous solution of AgNO_3_ (1 × 10^−3^ M) at 4 °C. During this stage, the silver ions were quickly absorbed and AgNPs were progressively formed. The hydrogel disk color changed nearly instantly from transparent to light yellow, which turned into brown color within 3 h, and finally evolved to dark brown within 10 h. Then, no color change was observed and reaction was stopped by replacing the solution by fresh Milli Q water. To study the influence of Ag precursor concentration on AgNPs formation in PU hydrogels, the above procedure was employed, embedding hydrogel disks in solutions of 5 × 10^−4^, 1 × 10^−4^, 5 × 10^−5^, and 1 × 10^−5^ M AgNO_3_.

Both Ag-hydrogel nanocomposite types were characterized using a combination of transmission electron microscopy (TEM) and UV-Vis extinction spectroscopy. UV-visible extinction spectroscopy analysis of the localized surface plasmon resonance (LSPR) and cloudy point measurements were carried out in a Cary 3 BIO-Varian UV-visible spectrophotometer (Palo Alto, CA, USA) equipped with a Peltier temperature control device. The transmission electron microscopy (TEM) was used to find out the size of AgNPs inside the hydrogel nanocomposite. To image the AgNPs, the hydrogel was cut into a number of very small pieces and incorporated within LR-White resin (London Resin Co. Ltd., Basingstoke, Hampshire, UK) for posterior ultrathin sectioning (Ultracut Reichert-Jung, Austria). 90 nm ultrathin sections were finally collected on Formvar-coated copper grids and observed with a field emission scanning electron microscope (FE-SEM) (Hitachi, SU 8000, Tokyo, Japan) at 30 kV using scanning transmission electron microscope (S-TEM) detector to detect the transmitted electrons.

### 2.4. Equilibrium Swelling Values as Function of Temperature

Equilibrium swelling values at different temperatures were determined gravimetrically. Gel disks were left to swell in water solutions for 24 h to achieve equilibrium at each temperature. At regular intervals, the swollen gels were taken out, wiped superficially with filter paper, weighed until equilibrium was attained, and placed again in the same immersion bath. Data from the swelling studies are usually expressed in terms of water uptake, defined as the weight of water absorbed by the sample per unit weight of dry polymer [33]. All measurements were performed in triplicate and averaged.

### 2.5. Antibacterial Activity

Sensitivity of *Pseudomonas aeruginosa (ATCC 25922)* and *Escherichia coli (ATCC 2785)* to the different silver-hydrogel nanocomposites was tested using disk diffusion test. The cells were reactivated from stocks culture in Mueller & Hinton (Difco Laboratories, Detroit, MI, USA), after 24 h, the samples of *P. aeruginosa* and *E. coli* were placed in 0.9% saline solution. The concentration of the bacteria was controlled to 10^6^ CFU/mL and they were seeded in Mueller & Hinton culture medium using a sterile spreader under sterile conditions. Prior to the experiment, uniform 6 mm diameter disks were washed by soaking each of them in 3 mL of MilliQ water. Afterward, they were placed on the culture plate. The plates were then incubated at 37 °C for 24 h. To study the sustained effect, every 24 h, the disks were changed to a new agar plate in which the bacterial suspension was previously spread. Inhibition zone diameters were measured in millimeters for each specimen. Commercially available, antimicrobial susceptibility test disks containing ceftazidime (30 μg, Oxoid, Columbia, MD, USA)) were used for positive control experiments of the antimicrobial effect. Assays were performed in triplicate and the data are shown as the mean ± standard deviation (SD).

## 3. Results and Discussion

### 3.1. Hydrogel Characterization

ATR-FTIR measurements were realized to corroborate the chemical structure of the hydrogel networks, via the identification of specific functional groups; and the typical spectra for dried thiol-acrylate (PSA) and polyurethane (PU) hydrogels, (Figures S1 and S2 of Supplementary Information SI, respectively), shows their achievement. In fact, Figure S1 exhibits the vibration bands associated with the expected PSA-hydrogel functional groups generated via thiol-ene radical polymerization (Scheme 1a). However, it is worth noting that even when no free thiol group signals can be observed, the presence of acrylate double bond vibration band at 1640 cm^−1^ denotes that not all the acrylate groups reacted. A plausible explanation of such behavior can be found in the known competitive chain- and step-growth reaction mechanism directly related to the thiol/acrylate ratio [32]. Typical morphology of pore structure of the formed hydrogels is presented in Figure S3.

With regard to the other hydrogel system, the absence of the band at 2270–2285 cm^−1^, associated to the isocyanate groups (Figure S2), supports the successful formation of the PU hydrogel network via policondensation [31] (Scheme 1b).

The thermal properties of these hydrogels, in terms of degradation temperature (*T*_max_) and glass transition (*T*_g_), were evaluated by TGA and DSC, respectively. The comparative data concerning thermal degradation values are collected in Table 1. From Table 1 it can be seen that each hydrogel system presents a unique degradation process, *T*_max_ , in the range of 350 to 400 °C with quite similar thermal stability in all cases and also similar to PEG linear polymers [34]. However, the slightly higher values for thiol-acrylate polymer based hydrogels compared with the data corresponding of polyurethane polymer based ones indicate that PSA hydrogel-related structure could still provide better thermal stability. Quite similar *T*_g_ values for hydrogels containing 100%-mol PEG (PSA100 and PU100, Table 1) were observed, in accordance to the values (about −45 °C) reported in the literature for linear PEG_600_/HDI Pus [31,35]. However, increases of PCL content confer a *T*_g_ diminution in the thiol-acrylate hydrogels (PSAs), that can be attributed to the molecular weight differences between the two polymers (PCL, 10 kDa; PEG, 3.35 kDa) involved in the polymerization. Basically, a higher PCL molecular weight would exert an increase of the length between crosslinking points. No similar tendency was observed for the PU-based hydrogels. Actually, glass transition exhibits no dependence on composition since PEG and PCL segments, involved in these hydrogels structure, have similar lengths. The glass transition and thermal stability values for Ag–PSA and Ag–PU nanocomposites show that these properties are not affected by the presence of AgNPs through the polymer matrix.

### 3.2. Swelling as a Function of Temperature

To understand and control the properties of hydrogel, structural parameters, such as crosslinking density and hydrophilicity of the polymer network that form the hydrogel, need to be taken into consideration. One important characteristic of hydrogels is the swelling response upon contact with water to an equilibrium disk thickness [33].

**Figure 1:**
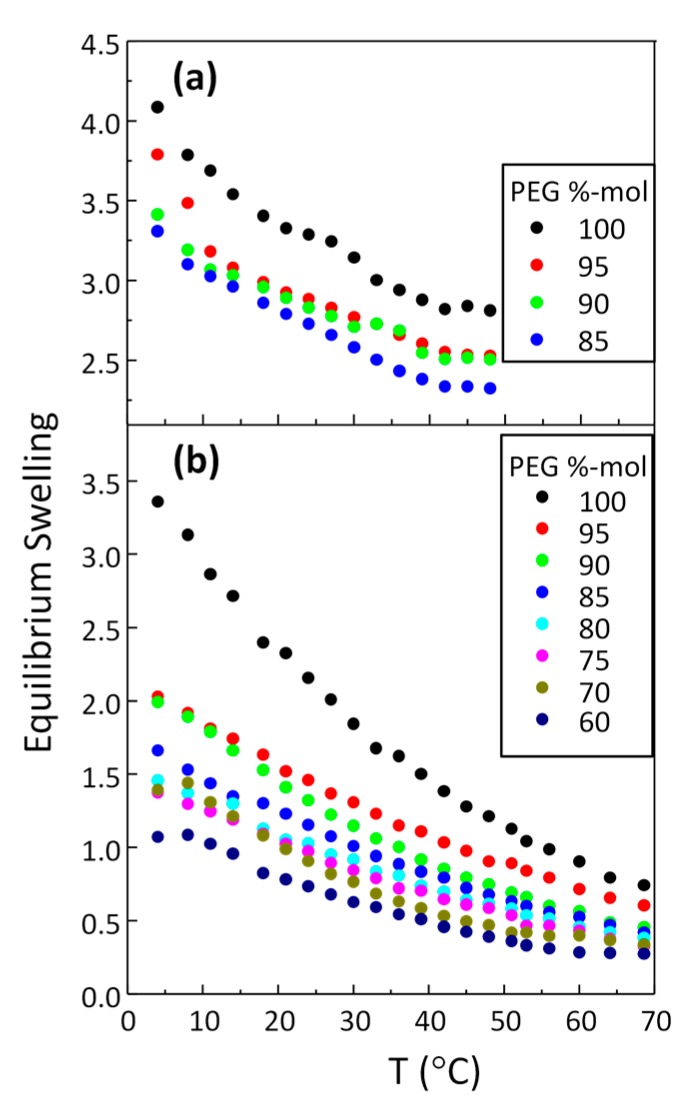
Equilibrium swelling degree of thiol-acrylate (PSA) (**a**) and polyurethane (PU) (**b**) hydrogels as a function of temperature and of composition.

Consequently, volume phase transition is caused by a change in the hydrogel’s water content. In this regard, Figure 1 depicts the swelling ratio of PSA and PU hydrogels plotted as function of PEG composition and temperature. As can be extracted from Figure 1 comparison, the PSA hydrogels (Figure 1a) lead to a higher water uptake and larger swelling ratios. As far as the hydrophilicity is concerned, a higher hydrophobic character of PU hydrogels due to HDI segments content (Scheme 1b) leads to a lower swelling ratio (Figure 1b). Therefore, a clear dependence of the equilibrium swelling on the composition of the hydrogel is stated.

Another important behavior extracted from Figure 1 is the negative thermo-sensitivity of both hydrogels, since the equilibrium of swelling clearly depends on temperature. Hydrogels composed of PEG can abruptly change their physico-chemical properties in response to external stimuli, such as temperature [36,37], undergoing a phase separation at a critical temperature. This volume phase transition temperature (VPTT) is intrinsically linked to the chemical nature of the polymer backbone and can be tuned by polymer characteristics, such as molecular weight [36] and the PEG end groups [37]. In this sense, Figure 1 shows that in all cases, the higher the temperature of solution is, the lower the degree of swelling and solubility is. Figure 1 also reveals a more noticeable volume transition phase decrease with increasing temperature for PU hydrogels than for PSA hydrogels. Again, the balance between hydrophobic and hydrophilic polymer chains inside hydrogel networks must be invoked. Actually, Figure S4 of SI shows a change from transparent (swollen state) to opaque when polymer chains collapse due to hydrophobic chain dominance. Similar behaviors were reported for linear [35] and crosslinked [31] polyurethanes comprised by PEG and HDI. Actually, both temperature sensitive hydrogels exhibited a tunable cloud point or volume transition temperature via control of hydrophilic/hydrophobic balance. Thus, increases of hydrophilic PEG molecular weight [35] or incorporation of hydrophobic PCL chains [31] raise cloud point and volume transition temperature, respectively.

### 3.3. Silver/Hydrogel Nanocomposites

**Scheme 2:**
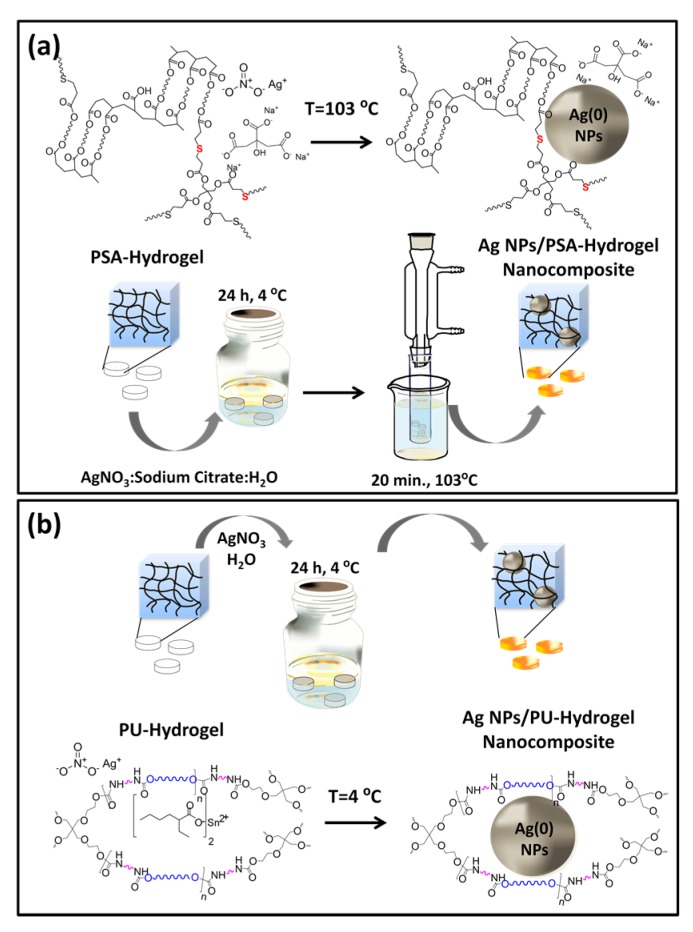
Schematic illustration portraying the formation of silver nanocomposites: AgNPs/PSA-hydrogel (**a**) and AgNPs/PU-hydrogel nanocomposites (**b**). Amphiphilic AgNPs were formed by in situ reactive-reduction process within preformed 3D-hydrogel matrices: thiol-acrylate (PSA) (**a**) and polyurethane (PU) (**b**) hydrogels.

To get a controlled nanostructured dispersion of Ag inside the two hydrogels, an in situ reactive nanoparticle formation within a preformed hydrogel-approach was considered. A schematic illustration of the two procedures is given in Scheme 2.

#### 3.3.1. AgNPs within Thiol-Acrylate Hydrogels (Ag–PSA)

3D PSA-hydrogel network, containing thiol reactive groups (Scheme 1a), was set up via crosslinking radical photo-polymerization (see Section 2.2 in Experimental) to act as template for the well-spacing growth of AgNPs into its structure (Scheme 2a). Then, nanoscale silver formation was conducted by soaking accurately weighed PSA hydrogel disks in an aqueous solution of AgNO_3_ and sodium citrate for a period of 24 h at 4 °C. During this incubation time, the hydrogel underwent appreciable swelling but no AgNP formation was observed. Afterward, glass tubes containing the silver salt absorbed hydrogels absorbed in the precursor solution were transferred into a silicon oil bath at 103 °C and allowed for 30 min to reduce the silver ions to AgNPs. A dark brown color in Figure S5 of SI indicates formation of AgNPs in hydrogel matrix. It is noteworthy that, during the exchange process of the silver ions from solution to hydrogel, the occupancy of free-network spaces of hydrogel is modulated by thiol-silver complexes formation in the presence of sodium citrate reductant. In this way, after temperature activation, an almost uniform distribution of AgNPs might be achieved within the hydrogel. Note that thiol groups exhibit high affinity to both Ag^+^ ions and colloidal Ag. Nevertheless, Figure S5 also reveals AgNP formation in the water outside the hydrogel. The explanation might be found in the key role played by hydrophobic/hydrophilic balance on the swelling degree of hydrogel over the temperature.

**Figure 2:**
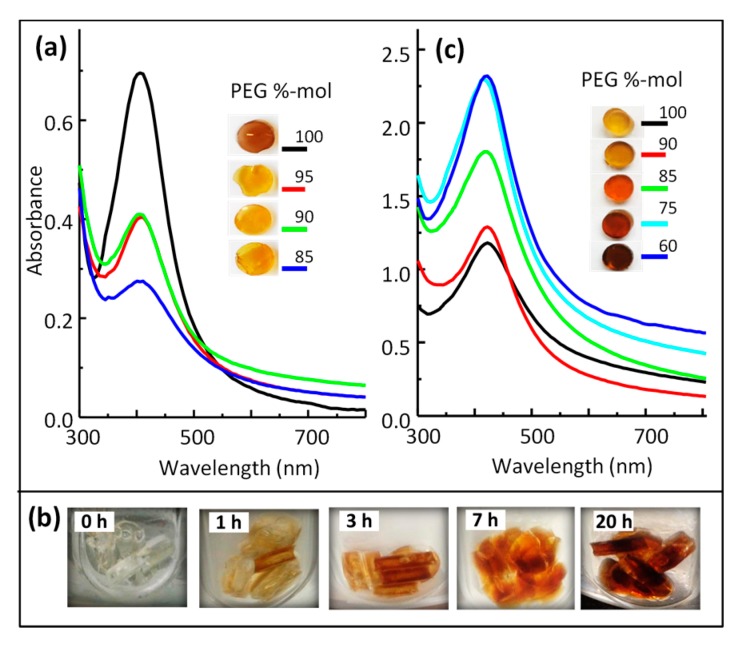
UV-Vis spectra of (**a**) Ag–PSA and (**c**) Ag–PU hydrogel nanocomposite disks for several PEG polymer compositions (%-mol), (Table 2). Photographs of the resulting Ag–PSA and Ag–PU hydrogel nanocomposite disks (Inset of (**a**) and (**c**) respectively). Photographic images following the AgNPs formation inside PU hydrogel matrices during the course of the reaction (**b**). The PU hydrogel disks were soaked in AgNO_3_ aqueous solutions at 4 °C.

Figure 2a shows the photographic images of the resulting Ag–PSA hydrogel nanocomposite disks. As it can be observed, the color intensity strongly depends on polymer composition, decreasing color intensity for copolymers with higher PCL content. This behavior could be explained by considering that the increased presence of PCL chains in the hydrogel copolymeric architecture leads to an increment of the hydrophobicity of the network, thus decreasing the swelling degree. In this context, another important factor to take into consideration is the swelling degree diminution with temperature, which undoubtedly leads to lower particle formation inside the hydrogels. In fact, the strong color of the soaking gel solution indicates that most of the AgNPs are formed outside the hydrogel network disks (Figure S5).

#### 3.3.2. AgNPs within Polyurethane Hydrogels (Ag–PU)

As mentioned in the introduction, an improved approach from the loading nanoparticle precursors into a gel was considered for building up Ag–PU hydrogel nanocomposites. Interestingly, AgNPs were formed in water solution not only in the absence of sodium citrate but even with a reaction temperature of 4 °C. The strategy based on the redox chemistry of the catalyst was involved in the PU hydrogel network-assembly. Residual stannous ethylhexanoate amount strategically acts as a reducing agent of silver precursor (AgNO_3_) to nanoparticles, so that nanoparticle-hydrogel composites are formed in the absence of additional external reducing agent.

Therefore, copolymer PU hydrogel was previously generated by condensation polymerization, and subsequently used as nanoreactor for silver nanoparticle formation (Scheme 2b). The formation of the silver nanoparticles into the hydrogel structure was then performed by contacting hydrogel with AgNO_3_ in deionized water solution at 4 °C. During this stage, the fast silver solution absorption derived on progressive AgNP formation, due to the oxidation/reduction reaction between the silver salt and stannous ion in the hydrogel copolymer-network (disk). The color of the hydrogel evolved from light yellow to dark brown. The snapshots, displayed in Figure 2b, demonstrate this nanoparticle generation. As it can be observed, a fast increase of yellowish color indicates almost immediate nanoparticle formation (0–30 min), which turned then to deep brown color (around 3–7 h) and remained stable after 12 h.

It is worth noting that, contrary to what happens to PSA hydrogels—where hydrogel surrounding the solution exhibits AgNP presence (Figure S5)—PU hydrogels only have particles forming inside the hydrogel disks. In analogy to the reported in the literature [38,39,40], stannous compound acts as reducing catalyst, which promotes AgNPs formation through AgNO_3_ reduction. So, during the reduction process, Sn^2+^ oxidizes into Sn^4+^ which reduces Ag^+^ into Ag°, and AgNPs are formed in a high yield. To the best of our knowledge, this is the first report about the in situ formation of AgNPs in polyurethane gels, taking advantage of remaining stannous ethylhexanoate catalyst. The reported references [38,39,40] involve AgNPs formation in organic solvents using stannous acetate as reducing agent and silver acetate as Ag precursor at higher temperature. Therefore, the low temperature reaction (4 °C) and the aqueous solution as reaction media make the main advantages of our system evident.

### 3.4. Optical Properties of Silver/Hydrogel Nanocomposites

UV-Vis absorption spectroscopy was carried out to monitor the AgNPs formation and gain insight into the particle size and shape. All UV-Vis traces (Figure 2a,c) showed an intense absorption band near 400–420 nm, depending on sample composition, characteristic of spherical AgNPs. This band, known as ‘surface plasmon resonance’ (SPR), is sensitive to geometric parameters (size and shape), aggregation, and the surrounding matrix of metal NPs. Hence, the plasmon band intensity is related to nanoparticle concentration, whereas the absorption maximum wavelength and shape is associated with nanoparticle size and aggregation.

Figure 2a,c displays the SPR band for our hydrogel nanocomposite systems (Ag–PSA and Ag–PU, respectively) synthesized. From mere inspection of figures, it follows that the peak intensity clearly decreases (Figure 2a) and increases (Figure 2c) with increasing PCL content; as result of the opposite contribution that the hydrophobic PCL segments exert on the silver nanoparticles genesis in both hydrogel systems. Moreover, this chemical composition dependence seems to be more noticeable for PSA hydrogels (Figure 2a); since approximately three-fold higher peak intensity diminutions were obtained as compared to the found intensity variations for PU hydrogels (Figure 2c). In fact, 50% and 75% peak intensity reductions were observed by simple increment of PCL content from 5% up to 10%-mol; whereas increased values of only 15% and 20% peak intensity were reported for similar PCL %-mol contribution in PU hydrogels (Table 2). These results demonstrate a clear shrinkage contribution of hydrophobic PCL chains in the swelling behavior of Ag-PSA hydrogel nanocomposites, which leads to a lower AgNP generation at 103 °C.

Furthermore, if we compare the absolute values of the peak intensities for equivalent PCL %-mol contributions of the both types of hydrogel nanocomposites (Ag–PSA and Ag–PU, Table 2), it results in the remarkable formation of higher AgNP concentration for PU hydrogels (Figure 2c), almost independently of their polymer composition. The explanation of these differences might be found in the two different approaches used by in situ reducing of silver nitrate in the hydrogel network. So, while PU hydrogel absolutely contains reduction agent inside hydrogel network, PSA hydrogel guarantees its presence through its dynamic diffusion equilibrium between the surrounding media and the inside of hydrogel. Therefore, NPs in the PSA system will be formed inside and outside the hydrogel framework (disk), and subsequently lower NP concentration (peak intensity) will be observed by UV measurement (Figure 2a and Table 2).

With respect to PU system, the NPs formation follows a process markedly driven by the stannous catalyst; so the chemical composition of the PU hydrogel should preferably influence the NP formation rate rather than the final NP concentration. In spite of this fact, PCL chains contribute, at some extent, in the process. Indeed, they act as: (i) stannous catalyst traps on their hydrophobic domains, leading to an improved SPR intensity effect with PCL content increase; and (ii) a lowered water uptake promoter, as a result of the much lowest swelling capacity of PU hydrogels (Figure 1), which ends up as a fictional SPR intensity increase appearance. These effects might explain the increased tendency of SPR intensity with increasing hydrophobic chain (PCL) content (Figure 2c); somehow inferred from dissimilarities on swelling degree capacities of hydrogels.

**Figure 3:**
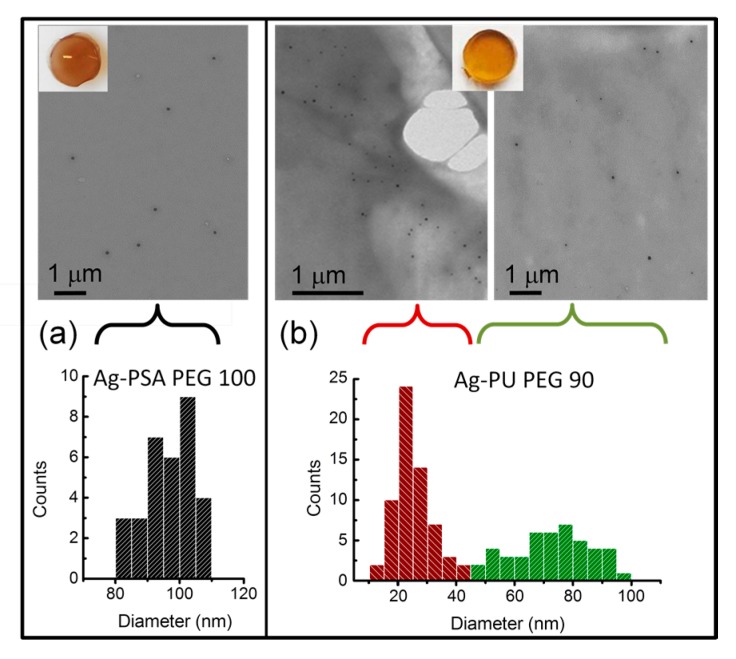
Representative bright field S-TEM images (upper) and respective five-bin histograms showing the size-distribution of AgNPs (below) prepared in Ag–PSA PEG 100 (**a**) and Ag–PU PEG 90 (**b**) hydrogel nanocomposites. Insets reproduce the light photograph of each Ag-hydrogel nanocomposite. Polyurethane-based nanocomposites show two different NP size-populations (25 ± 8 nm and 73 ± 13 nm right representations).

The optical properties of both hydrogel nanocomposites were further investigated by variable SPR band shape. The band shape symmetry and the lack of peak shift (Figure 2a,c) indicate that the spherical AgNPs were well dispersed and not forming aggregated structures. S-TEM image of Ag–PSA and Ag–PU hydrogel nanocomposites (Ag–PSA PEG 100 and Ag–PU PEG 90, respectively in Table 2) mostly reveals AgNPs with uniform dimension and morphology (Figure 3). Sizes of 97 ± 8 nm for Ag–PSA PEG 100 were estimated for an overall of 50 nanoparticles counted (Figure 3a). However, direct observation of Ag–PU hydrogel nanocomposite samples (Table 2) by means of several S-TEM photographs evidenced two populations of nanoparticles: one of small size (25 ± 8 nm) and another with a different size around 73 ± 13 nm. Figure 3b displays representative S-TEM images of Ag–PU PEG 90 hydrogel nanocomposite to visualize these two different NP size-populations.

Interestingly, from Figure 2c slight asymmetric broadening of the SPR band toward shorter wavelengths with increasing hydrophobic chain content can be inferred. Complementary information to that in Figure 2c is obtained by inspection of the signal intensity and position collected from SPR bands of a silver-polymer hydrogel with PEG 60%-mol (Ag-P 60*) nanocomposite sample in Figure S6. Basically, the Ag-P 60* hydrogel matrix sample was performed in the absence of PEG_300_ crosslinker and with similar PCL and PEG %-mol contents (Scheme 1b) as those of Ag–PU PEG 60 (see Section 2.2 in Experimental). By this protocol, the potential to dissolve a physically crosslinked 3D-hydrogel structure under basic conditions in ethanol was obtained. The resulting un-crosslinked 3D-polyurethane hydrogel matrix (P 60*) was then used to prepare AgNPs within, following the procedure described in Section 2.3 of the experimental part. Interestingly, UV-Vis spectra (Figure S6) display a unique SPR band for the both Ag–PU nanocomposites (gel-disk (Ag–PU PEG 60, red line) and gel-solution (Ag-P 60*, green line) sample) evidencing the cooperative behavior of the two NP size populations; which only splits into two SPR bands when the enriched fraction on the smallest NPs (supernatant SN-Ag-P 60*)—as obtained by low speed centrifugation of Ag-P 60* (gel-solution)—is measured (Figure S6, black line). Indeed, the appearance of a second peak toward blue end of the spectrum can be attributed to a different NP size-population, formed during the reduction process within the hydrogel, attesting to the two observed NP sizes in the S-TEM image of Figure 3b. Moreover, considering the pivotal NP surface charge effect in the osmotic pressure of hydrogel nanocomposites [41], we hypothesized that the initial content of immobilized charge (due to small silver colloids generated along the in situ reactive nanosilver-loading of PU hydrogels) would induce a hydrogel osmotic swelling, despite the opposite PCL effect. Then, at a higher surface charge, more nuclei are formed and result in the formation of another NP population with a different range of sizes inside the hydrogel scaffold. Note that small NPs have a greater surface area at a fixed NP concentration; and a more effective surface charge, subsequently. It is worth pointing out though that longer SPR wavelengths are obtained over PU-based Ag-nanocomposites than over PSA-based ones, as depicted from Figure 2a,c comparison; mainly attributed to differences on the surrounding refractive index of both systems. Note that AgNPs within PSAs are protected by sodium citrate, whereas within PUs must be covered by the polymer.

**Figure 4:**
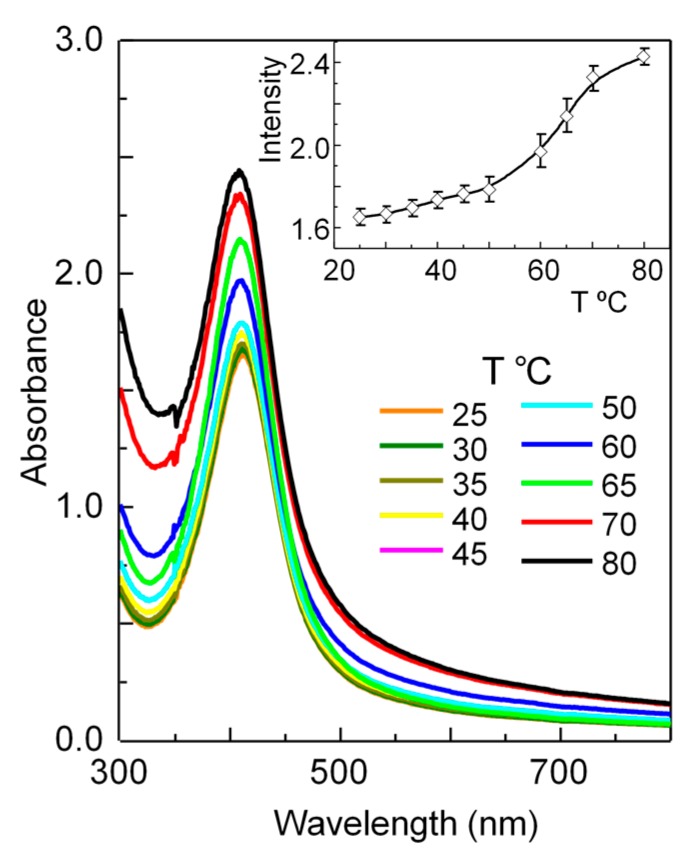
UV-Vis spectra of Ag–PU PEG 85 hydrogel nanocomposites when temperature increases from 25 to 80 °C. Inset: SPR band maximum intensity of Ag–PU PEG 85 as a function of the temperature.

Further investigations of the SPR band evolution at various temperatures (Figure 4) for Ag–PU nanocomposites containing 85%-mol PEG (Ag–PU PEG 85, Table 1) confirmed the effective dispersion of NPs inside the hydrogel.

Figure 4 unequivocally shows a strong plasmon intensity increase (about 50%), and no shift in the absorption profile, as a result of the volume phase transition of the hydrogel when the temperature rises to 50 °C; which may be ascribed to the surrounding polymer collapse on particles core surface. Indeed, PU hydrogel with 85% PEG exhibits a quite similar decrease of swelling equilibrium (around 57%) in between 25 and 50 °C, as displayed in Figure 1.

**Figure 5:**
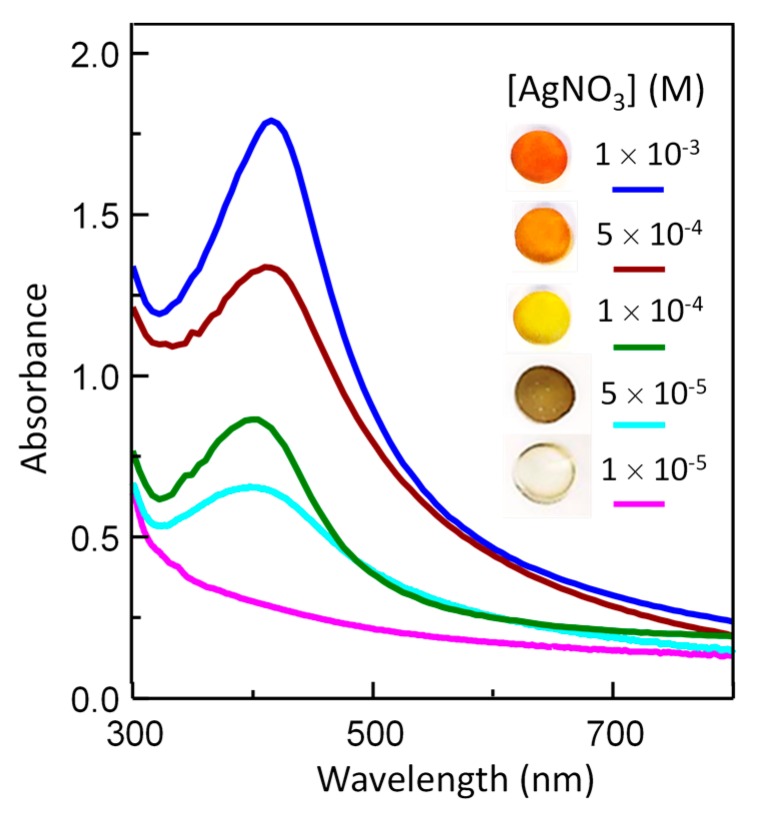
UV-Vis of spectra of Ag–PU PEG 85 hydrogel nanocomposite disks, while increasing the AgNO_3_ concentration added to the PU hydrogel disk (Table 3). Inset: Pictures of the resulting Ag–PU hydrogel nanocomposite disks (error bars represent standard deviation for studies conducted in triplicate).

An additional experiment concerning, now, the influence of Ag precursor concentration on AgNPs formation in the PU hydrogel was evaluated. UV-Vis spectra were recorded for Ag–PU hydrogel nanocomposites with a constant PEG content (85%-mol, Ag–PU PEG 85) but varying AgNO_3_ content (Table 3 and Figure 5). As expected, the number of AgNPs created was proportional to the AgNO_3_ content; a linear trend of increase of SPR maximum intensity with the increase of AgNO_3_ concentration was observed (Figure 5). Interestingly, a blueshift of SPR band and a yellowish color with Ag precursor content diminution (inset, Figure 5) argue favorably for the initial formation of small and discrete AgNPs. For 10^−5^ M AgNO_3_ concentration, no silver nanoparticle formation was observed.

### 3.5. Antimicrobial Properties

**Figure 6:**
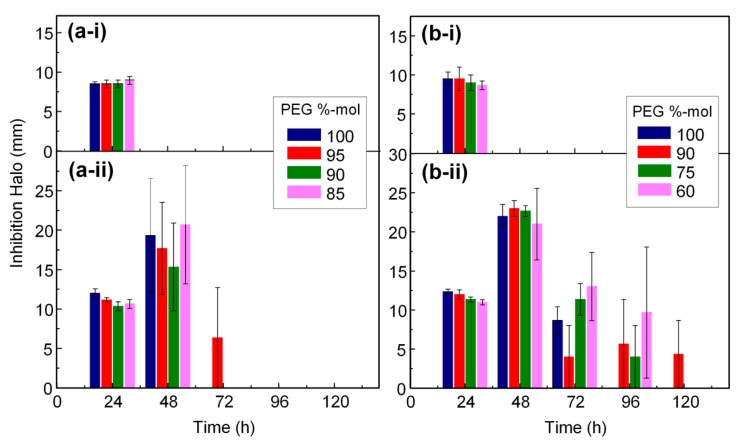
Graphical representation showing the antimicrobial efficacy of (**a**) Ag–PSA and (**b**) Ag–PU hydrogel nanocomposites: Inhibition halo distance (mm) over time against (i) *E. coli* and (ii) *P*. *aeruginosa*. Error bars represent standard deviation for studies conducted in triplicate.

Antimicrobial properties of Ag–PSA and Ag–PU hydrogel nanocomposites were evaluated against Gram-negative bacteria, *P. aeruginosa* and *E. coli*, using the disk diffusion test method. In all cases, these properties were measured by evaluating the zone of inhibition around the disk after incubation at 37 °C. With this aim, uniform disks with a 6 mm diameter and 1 mm thickness obtained from Ag–PSA and Ag–PU hydrogel nanocomposites, were placed on agar Petri dishes, where the bacterial suspension was previously spread (Figures S7 and S8, respectively). A simple observation of the photographs (Figures S7 and S8) revealed that the Ag-hydrogel nanocomposites exhibited antimicrobial properties. However, the hydrogel systems without Ag showed no zone of inhibition against *E. coli* and *P. aeruginosa.* A quantitative analysis of these results is reported in Figure 6a,b. Figure 6a shows measurements of the inhibition zone for Ag–PSA hybrids over time for *E. coli* (Figure 6a-i) and *P. aeruginosa* (Figure 6a-ii). Comparing the inhibition results after the first 24 h (Figure 6a), it appears that all Ag-nanocomposites were able to inhibit bacterial growth without copolymer composition dependence. A similar behavior was found for Ag–PU hydrogel nanocomposites in Figure 6b. Moreover, both hydrogel nanocomposite systems exhibited a more effective antibacterial activity against *P.* *aeruginosa* (Figure 6a-ii and 6b-ii) than against *E. coli* (Figure 6b-i), as proven with the higher inhibition halo. The explanation of this fact could lie on the ability of *E. coli* to develop heavy metal resistance, particularly for silver [1,42]. Remarkably, the inhibition halos after 24 h cultivation were slightly higher for PU hydrogel nanocomposites. Although the most interesting feature of our systems is the sustained antimicrobial activity against *P. aeruginosa* (Figure 6a-ii and 6b-ii). In fact, after 48 h incubation, the bacterial inhibition effect strongly increases, almost doubling for Ag–PU hydrogel nanocomposites. Polyurethane-based nanocomposites, possibly due to their higher AgNP content (Figure 2c and Table 2), exhibit a longer sustained effect (Figure 6b-ii). Values up to above 120 h incubation can be observed for samples containing 90%-mol PEG. We speculate that prolonged inhibition may be associated with the position and the accumulation of generated AgNPs through the hydrogel network (disk). So, a closer AgNP location to the contact surface between 3D hydrogel nanocomposites and culture medium, where the bacteria is spread, will induce an increased and sustained inhibition effect. The antibacterial efficacy of the hydrogel nanocomposites is determined by the size of nanoparticles within the polymeric structure. A bigger size of AgNPs in PSA hydrogels decreases the antibacterial effect, whereas the smaller size of AgNPs would increase antibacterial activity. The reason is because, with decreasing size, the total surface area per unit volume is greater; this phenomenon facilitates nucleation of a higher number of nanoparticles within the polymeric network [43,44]. The degree of swelling and cross-linking is also related to this effect. In the specific case of the PU hydrogels, the diffusion rate is slower than in the case of PSA hydrogels, whose rate promotes a faster diffusion, having a higher degree of swelling. This analysis demonstrates the sustained antibacterial effect until 120 h incubation for the PU hydrogel nanocomposites.

In addition, the presence of different NP sizes with larger antibacterial reactive surface areas as attested by UV-Vis (Figure S6) and confirmed by S-TEM (Figure 3) correlates well with an increased sustained effect of PU based Ag-nanocomposites.

Resistance—more by *E. coli* than by *P. aeruginosa*—may be due to the features of its cell membrane, which has properties of altering the permeability [45,46]. This defense mechanism is the most likely that limits the sustained antibacterial activity against *E. coli* strain. The analysis of antimicrobial activity by inhibition zone shows that the effect against studied bacterial strains is effective, which represents, in the future, great advantages in biomedical application.

## 4. Conclusions

In this work, we reported an easy and effective strategy to produce Ag hydrogel nanocomposites by in situ reactive-reduction of AgNPs within polymeric-hydrogel matrices. The system is based on biocompatible and biodegradable PSA and PU hydrogels, previously synthesized by thiol-acrylate radical polymerization and polyurethane chemistry. Then subsequently used as templates for the growth of AgNPs. A good dispersion and sharp-particle-size distribution of AgNPs is followed by simple loading of nanoparticle precursor (AgNO_3_)—in the presence and absence of an external reducing agent—within PSA and PU hydrogels respectively. Interestingly, Ag–PU hydrogel nanocomposites were newly obtained via redox chemistry of residual stannous catalyst from the polyurethane synthesis in the absence of temperature (4 °C); while thiol-silver ion affinities would facilitate some control over NP size and distribution in Ag–PSA hydrogel nanocomposites. We systematically investigated the swelling and optical behaviors of the both series of thermosensitive hydrogels on their in situ nanoparticle generation. It was shown that a higher contribution of hydrophobic segments in the hydrogel network induced a lower swelling response, which led to a lower NP content. Also, SPR shape and intensity as a function of the hydrogel chemical-composition revealed spherical and well-spaced AgNPs with non-aggregated nanoparticle-assembly evidence. Nevertheless, certain SPR asymmetry of Ag–PU hydrogel nanocomposites demonstrates the formation of two NP-populations, probably due to the increase of nucleation/growth rates generated by changes in osmotic swelling induced by surface charge on small NPs. S-TEM images confirm these data. Furthermore, the antimicrobial behavior of both Ag-loaded hydrogels showed growth inhibition of Gram-negative bacteria as *P. aeruginosa* and *E. coli*; suggesting a more effective Ag^+^ releasing hydrogel against *P. aeruginosa* strain. Remarkably, Ag–PU hydrogel nanocomposites exhibited sustained inhibition of *P. aeruginosa*, detectable even after 120 h cultivation. Therefore, these mechanisms emphasize the pivotal role of AgNP size and location in hydrogel matrix, in the efficacy and sustainability of antibacterial activity. This illustrates the potential of these hydrogels for application biomedical fields, such as smart healing dressings for burns and surgical wounds, tissue engineering scaffolds, and ophthalmic therapies.

## Figures and Tables

**Table 1:** Thermal properties of (PSA and PU) polymer hydrogels and (Ag–PSA and Ag–PU) silver hydrogel nanocomposites: maximum degradation temperature (*T*_max_), glass transition temperature (*T*_g_) and melting temperature (*T*_m_).

Hydrogel ^a^	*T*_max_ (°C)	*T*_g_ (°C)	*T*_m_ (°C)	Ag-Nanocomposites	*T*_max_ (°C)	*T*_g_ (°C)	*T*_m_ (°C)
PSA100	376	−45	43	Ag–PSA PEG 100	378	−46	44
PSA95	367	−47	44	Ag–PSA PEG 95	365	−48	46
PSA90	394	−51	42	Ag–PSA PEG 90	383	−52	44
PSA85	389	−53	41	Ag–PSA PEG 85	385	−52	43
PSA PCL	403	−58	54	Ag–PSA PCL	-	-	-
PU100	351	−44	-	Ag–PU PEG 100	376	−44	-
PU95	341	−42	-	Ag–PU PEG 95	-	-	-
PU90	360	−43	-	Ag–PU PEG 90	382	−45	-
PU85	356	−43	-	Ag–PU PEG 85	350	−43	-
PU80	364	−42	-	Ag–PU PEG 80	-	-	-
PU75	356	−43	-	Ag–PU PEG 75	351	−43	-
PU70	357	−41	-	Ag–PU PEG 70	-	-	-
PU60	357	−41	-	Ag–PU PEG 60	358	−43	-
PU–PCL	330	−35	-	Ag–PU PCL	-	-	-

^a^ PSA- and PU-hydrogel nomenclature is referred to the PEG %-mol content. Ag–PSA PCL and Ag–PU PCL denote to PCL 100%-mol content (PEG 0%-mol content).

**Table 2:** Characteristics of the surface plasmon resonance (SPR) band for Ag–PSA and Ag–PU hydrogel nanocomposites.

Sample	Temperature of Synthesis (°C)	SPR Maximum Wavelength (nm)	SPR Maximum Absorbance
Ag–PSA PEG 100	103	405.7 ± 0.6	0.83 ± 0.05
Ag–PSA PEG 95		409.2 ± 0.7	0.41 ± 0.03
Ag–PSA PEG 90		406.2 ± 0.7	0.25 ± 0.02
Ag–PSA PEG 85		406.5 ± 0.8	0.37 ± 0.02
Ag–PU PEG 100	4	419.9 ± 0.8	1.02 ± 0.05
Ag–PU PEG 90		421.3 ± 1.1	1.22 ± 0.04
Ag–PU PEG 85		416.8 ± 0.6	1.61 ± 0.06
Ag–PU PEG 75		416.1 ± 0.9	1.60 ± 0.05
Ag–PU PEG 60		418.7 ± 0.9	1.75 ± 0.05

**Table 3:** Influence of AgNO_3_ concentration on the SPR band (position and intensity) of PU hydrogel with 85%-mol of PEG (Ag–PU PEG 85, Table 2).

Sample	AgNO_3_ (M)	SPR Maximum Wavelength (nm)	SPR Maximum Absorbance
Ag–PU PEG 85-1	1 × 10^−3^	416.8 ± 0.6	1.61 ± 0.06
Ag–PU PEG 85-2	5 × 10^−4^	412.9 ± 0.4	1.15 ± 0.05
Ag–PU PEG 85-3	1 × 10^−4^	400.8 ± 0.7	0.64 ± 0.03
Ag–PU PEG 85-3	5 × 10^−5^	397.7 ± 0.5	0.52 ± 0.02
Ag–PU PEG 85-3	1 × 10^−5^	-	0

## Supplementary Materials

The following are available online at http://www.mdpi.com/2079-4991/7/8/209/s1, Thiol-acrylate Hydrogel characterization by ATR-FTIR (Figure S1) and SEM (Figure S2); Polyurethane Hydrogel characterization by ATR-FTIR (Figure S3); UV-Vis spectra of different AgNP sizes formed within PU hydrogels (Figure S4); AgNPs generation outside-inside PSA hydrogels (Figure S5); Comparative UV–Vis spectra of Ag-PU PEG 60 (gel-disk, dissolved gel solution and supernatant solution obtained by centrifugation) (Figure S6); and Photographs of sustained antibacterial activity of Ag–PSA and Ag–PU hydrogel nanocomposites (Figures S7–S9).
