# Supplementary material for: 3D-Hydrogel Based Polymeric Nanoreactors for Silver Nano-Antimicrobial Composites Generation
Source: Nanomaterials (Basel). 2017 Aug 1;7(8):209. doi: 10.3390/nano7080209 (PMC5575691; doi:10.3390/nano7080209)
Supplement: Supplementary file 1 [file nanomaterials-07-00209-s001.pdf]

Supplementary Information

3D-Hydrogel Based Polymeric Nanoreactors for Silver Nano-Antimicrobial Composites Generation

Albanelly Soto-Quintero [1], Ángel Romo-Uribe [2] and Víctor H. Bermúdez-Morales [3], Isabel Quijada-Garrido [4,*] and Nekane Guarrotxena [4,*]

1. Centro de Investigación en Ingeniería y Ciencias Aplicadas, Universidad Autónoma del Estado de Morelos, Cuernavaca 62209, Morelos, México; soquia_17@hotmail.com
2. Research & Development, Advanced Science & Technology Division, Johnson & Johnson Vision, FL 32256, USA; aromouribe@gmail.com
3. Centro de Investigación sobre Enfermedades Infecciosas. Instituto Nacional de Salud Pública. Dirección de Infecciones Crónicas y Cáncer, Avenida Universidad No. 655, Cerrada los Pinos y Caminera, Colonia Santa María Ahuacatitlán, Cuernavaca 62100, Morelos, México; vbermudez@insp.mx
4. Instituto de Ciencia y Tecnología de Polímeros, Consejo Superior de Investigaciones Científicas (ICTP-CSIC), c/ Juan de la Cierva, 3. E-28006 Madrid, Spain

* Correspondence: nekane@ictp.csic.es (N.G.); iquijada@ictp.csic.es (I.Q.-G.); Tel.: +34-915-622-900 (N.G.); +34-915-622-900 (I.Q.-G.)

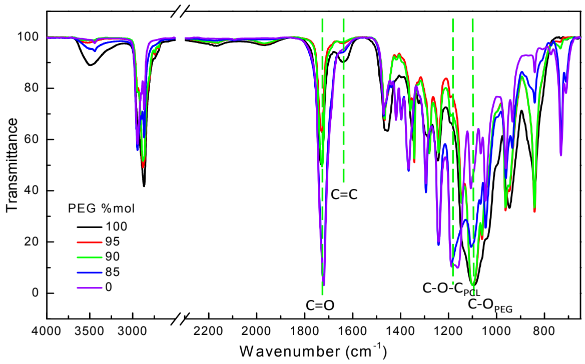

Figure S1. ATR-FTIR spectra of thiol-acrylate (PSA) hydrogels (Table 1)

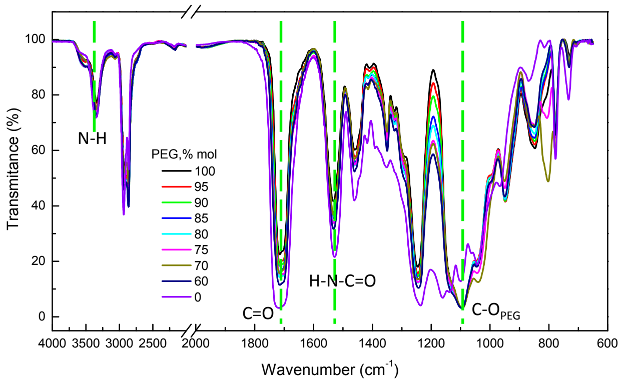

Figure S2. ATR-FTIR spectra of polyurethane (PU) hydrogels (Table 1)

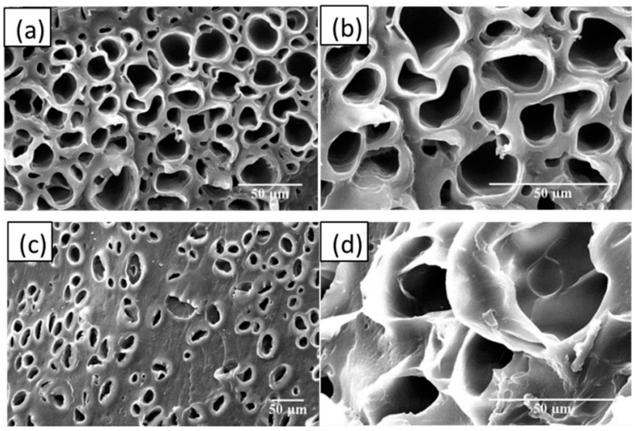

Figure S3. Representative SEM image showing the pore structure corresponding to PSA hydrogels at different magnification: (**a, b**) PEG 100%-mol content, and (**c, d**) PEG 95 %-mol content

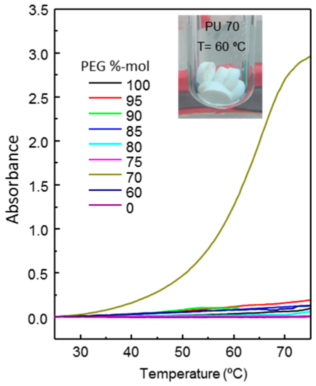

Figure S4. Changes of UV-Vis absorption band (at 600 nm) of polyurethane (PU) hydrogels (Table 1) as a function of temperature. Inset: Photograph of PU 70 hydrogel disks (PEG 70%-mol content, (Table 1) at temperatures higher than the volume phase transition temperature, 60 °C.

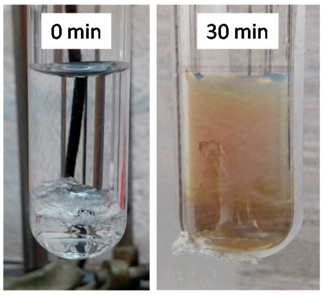

Figure S5. Visual observation of Ag–PSA hydrogel nanocomposites synthesis after 30 min. AgNPs formation outside hydrogel can also be perceived. PSA hydrogel sample contains 100%-mol of PEG (PSA 100).

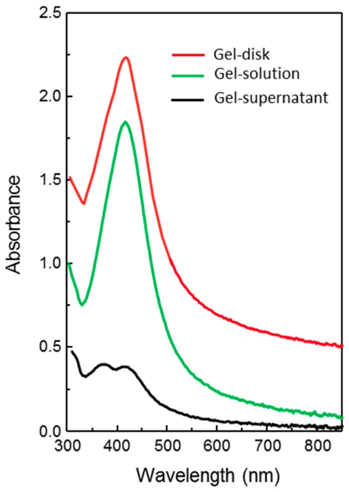

Figure S6. Comparative UV-Vis spectra of Ag-PU PEG 60 (gel-disk, red line), Ag-P 60* (gel-solution, green line), and SN-Ag-P 60* nanocomposites, enriched fraction on the smallest NPs, as obtained by low speed centrifugation of the Ag-P 60* sample (supernatant of gel-solution, black line).

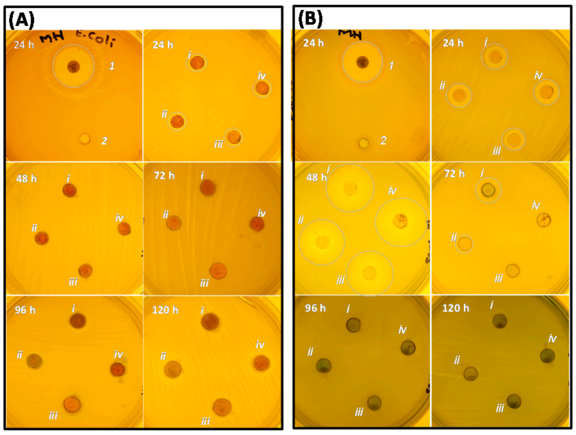

Figure S7. Photographs showing the sustained antibacterial activity of Ag–PSA hydrogel nanocomposites with different PEG contents (i) PEG 100%-mol, (ii) PEG 95%-mol, (iii) PEG 90%-mol and (iv) PEG 85%-mol against (A) *E. coli* and (B) *P. aeruginosa*. Zone of inhibition produced by Ag–PSA nanocomposites against the two tested bacterial strains after 24, 48, 72, 96 and 120 h. Test disk of Ceftazidime 30 µg was used as a positive control (1) compared with a blank disk (2).

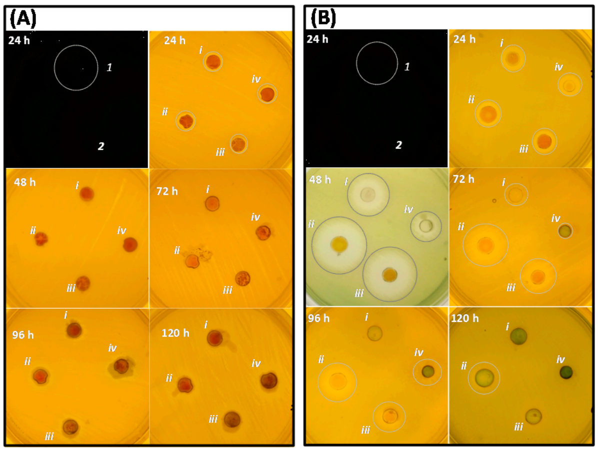

Figure S8. Photographs showing the sustained antibacterial activity of Ag–PU hydrogel nanocomposites with different PEG contents (i) PEG 100%-mol, (ii) PEG 90%-mol, (iii) PEG 75%-mol and (iv) PEG 60%-mol against (A) *E. coli* and (B) *P. aeruginosa.* Zone of inhibition produced by Ag–PU nanocomposites against the two tested bacterial strains after 24, 48, 72, 96 and 120 h. Test disk of Ceftazidime 30 µg was used as a positive control (1) compared with a blank disk (2).

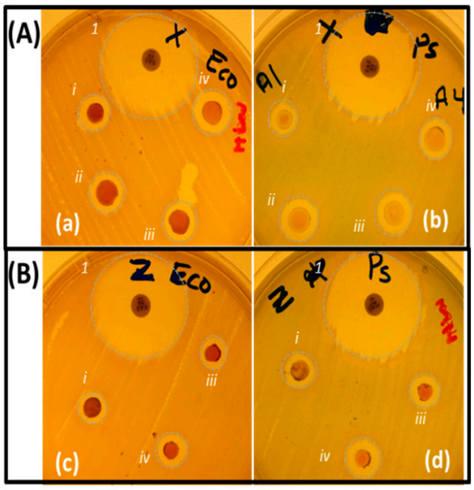

Figure S9. Comparative photographs showing the zone of inhibition produced by: (**A**) Ag–PSA hydrogel nanocomposites with (i) PEG 100%-mol, (ii) PEG 95%-mol, (iii) PEG 90%-mol and (iv) PEG 85%-mol against (**a**) *E. coli* and (**b**) *P. aeruginosa;* and (**B**) Ag–PU nanocomposite hydrogels with (i) PEG 100%-mol, (iii) PEG 75%-mol, (iv) PEG 60%-mol against (**c**) *E. coli* and (**d**) *P. aeruginosa*. Control: Ceftazidime 30 µg (1). The plates were incubated at 37 °C for 24 h.
